# Five new species of Minettia (Minettiella) (Diptera, Lauxaniidae) from China

**DOI:** 10.3897/zookeys.449.7807

**Published:** 2014-10-22

**Authors:** Li Shi, Ding Yang

**Affiliations:** 1 College of Agronomy, Inner Mongolia Agricultural University, Hohhot, Nei Mongol 010019, China; 2 Department of Entomology, China Agricultural University, Beijing 100193, China

**Keywords:** *Minettiella*, synonym, female terminalia, Oriental region

## Abstract

Five species of *Minettia* Robineau-Desvoidy, 1830 from the South of China are described as new to science: Minettia (Minettiella) bawanglingensis **sp. n.**, Minettia (Minettiella) clavata **sp. n.**, Minettia (Minettiella) plurifurcata **sp. n.**, Minettia (Minettiella) spinosa **sp. n.** and Minettia (Minettiella) tianmushanensis **sp. n.** *Minettiella elbergi* Shatalkin, 1996 and *Minettia japonica* Sasakawa, 1995 are treated as junior synonyms of Minettia (Minettiella) dolabriforma (Sasakawa & Kozánek, 1995). A key to five subgenera of *Minettia* and eleven species of Minettia (Minettiella) is presented.

## Introduction

The subgenus *Minettiella* Malloch, 1929, of the genus *Minettia* Robineau-Desvoidy, 1830, was erected for the type species *Lauxania atratula* Meijere, 1910, being glossy black, and having immaculate wings, yellow halteres, a katepisternum with a discal katepisternal seta, pubescent aristae, shining frons, a flat face, and mesonotum with 0–1+2–3 dorsocentral setae and 0–1+2–4 acrostichal setae, and most species having one to three pairs of strong acrostichal setae (Malloch 1929; Stuckenberg 1971). Actually, all species have pruinosity on the mesonotum and abdomen (only the frons is shining black), so the so-called “glossy” black habitus is not correct.

Shatalkin (1996) had elevated the subgenus *Minettiella* to the genus level when he described *Minettiella elbergi* Shatalkin, 1996. Shatalkin (2000) discussed that its having genus rank was based on the specialized male genitalia and the above-mentioned diagnosis, and noted that the described species *Minettiella acrostichalis* (Sasakawa & Kozánek, 1995) was possibly a synonym of *Minettiella coracina* Shatalkin, 1993, and two described species *Minettiella elbergi* and *Minettia japonica* (Sasakawa, 1995) were possibly synonyms of *Minettiella dolabriforma* (Sasakawa & Kozánek, 1995) (Shatalkin 2000). We examined the male genitalia of some specimens from five subgenera of *Minettia* and found that the diversity of the male genitalia (that is, there are lots of many different forms among the species) exists in the three subgenera *Minettia*, *Minettiella* and *Plesiominettia* Shatalkin, 2000, which is more diversity of male genitalia than that in two subgenera *Frendelia* (Collin, 1948) and *Scotominettia* (Shatalkin, 2008) based on many examined specimens of five genera from the Palaearctic and Oriental regions. The diversity of the male genitalia in *Minettiella* is the same as that in *Minettia* and *Plesiominettia*. So we reject Shatalkin’s elevation of the subgenus *Minettiella* to the genus level and consider *Minettiella* as a subgenus *Minettiella*. Moreover, we compared the male genitalia Minettia (Minettiella) elbergi and Minettia (Minettiella) japonica and determined them to be junior synonyms of Minettia (Minettiella) dolabriforma. The color of the mid and hind tibiae and the ratio of height and length of the 1st flagellomere in Minettia (Minettiella) coracina Shatalkin are distinctly different from that in Minettia (Minettiella) acrostichalis (Sasakawa & Kozánek, 1995), so the species Minettia (Minettiella) coracina Shatalkin is considered to be a valid species.

The type specimen of Minettia (Minettiella) atrata (Meijere, 1910) was recorded in Jong (2000) as missing the male genitalia, but fortunately the description and diagnosis is adequate to separate it from other species.

In total, there are eleven known species of the subgenus *Minettiella* with six currently found in China (see Appendix for species checklist).

## Materials and methods

The general terminology follows Gaimari and Silva (2010). Genitalia preparations were made by removing and macerating the apical portion of the abdomen in cold saturated NaOH for 6 hours. After examination, they were transferred to glycerine for examination and stored in a microvial on the pin below the specimen. Specimens examined were deposited in China Agricultural University, Beijing, China (CAUC).

The following abbreviations are used: a–anterior seta(e), acr–acrostichal seta(e), ad–anterior dorsal seta(e), app–apical posterior seta(e), apv–apical ventral seta(e), av–anterior ventral seta(e), dc–dorsocentral seta(e), ia–intra alar, kepst–katepisternal seta(e), oc–ocellar seta(e), or–fronto-orbital seta(e), p–posterior seta(e), pd–posterior dorsal seta(e), prsc–prescutellar seta(e), pv–posterior ventral seta(e).

## Taxonomy

### Species descriptions

Unless otherwise specified, the following seven species described below are characterized as follows: Head, thorax and abdomen black. Frons wider than long and parallel-sided. Face and parafacial flat with dense whitish gray pruinosity. Ocellar triangle black; *oc* developed, shorter than anterior *or*, anterior *or* reclinate, shorter than posterior *or*. Arista black with yellow at base. Scutellum black, with brown grayish pruinosity. All femora black or blackish brown. Wing slightly yellow, hyaline. Halter yellow.

#### Minettia (Minettiella) atratula

Taxon classificationAnimaliaDipteraLauxaniidae

(Meijere, 1924)

Figs 1 – 5 , 31 –32

Lauxania atratula Meijere, 1924: 49. Type locality: Indonesia (Sumatra).
Minettia (Minettiella) atratula : Malloch 1929: 26. Shewell 1977: 190. Shatalkin 1996: 147. Jong 2000: 32.

##### Material examined.

2 ♂♂, 1♀ (CAUC), CHINA, Hainan: Ledong, Jianfengling National Natural Reserve, Tianchi, 800 m, 18. v. 2006, Hui Dong; 1♂ (CAUC), CHINA, Hainan: Ledong, Jianfengling National Natural Reserve, Tianchi, 800 m, 18. v. 2006, Gang Yao.

##### Diagnosis.

Mesonotum 1+3 *dc*, *acr* in 2 rows with 1+3 long *acr*. Fore tibia yellowish brown with yellow at base and black at apex, mid tibia yellow and hind tibia yellow with brown at apex; fore tarsi with basitarsus yellow on basal 3/4 and tarsomeres 2–5 brown, mid and hind tarsi with tarsomeres 1–2 dark yellow and tarsomeres 3–5 pale brown. Mid femur with 4 *a*.

##### Redescription.

MALE. Body length 3.3–3.5 mm, wing length 3.4–3.5 mm. FEMALE. Body length 3.8 mm, wing length 3.9 mm.

Head. Frons slightly concave with narrow yellow anterior margin. Gena about 1/4 eye height. Antenna scape and pedicel yellowish brown to brown, 1st flagellomere blackish brown with yellow at base and nearly 2.3 times longer than high; arista bare. Proboscis black with dark yellow at apex. Palpus black.

Thorax with brownish gray pruinosity. Mesonotum with 1+3 *dc*, *acr* in 2 rows with 1+3 long *acr* in front of *prsc* and one pair of *prsc* as long as 1st postsutural *dc*. Legs: fore tibia mostly yellowish brown with yellow at base and black at apex; mid tibia yellow and hind tibia yellow with brown at apex; fore tarsi with basitarsus yellow on basal 3/4 and tarsomeres 2–5 brown, mid and hind tarsi with tarsomeres 1–2 dark yellow and tarsomeres 3–5 pale brown. Fore femur with 4 *pv* and 6 *pd*, fore tibia with 1 long preapical *ad* and 1 short *apv*. Mid femur with 4 *a* and 1 *app*; mid tibia with 1 strong preapical *ad* and 1 strong *apv*. Hind femura with 1 preapical *ad*, and hind tibia with 1 weak preapical *ad* and 1 short *apv*. Wing with costa with 2nd (between R_1_ and R_2+3_), 3rd (between R_2+3_ and R_4+5_) and 4th (between R_4+5_ and M_1_) sections in proportion of 6.7:1:1, *r-m* at middle of discal cell; ultimate and penultimate sections of M_1_ in proportion of 1:1.1; ultimate section of CuA_1_ about 1/6 of penultimate section.

**Figures 1–5.**
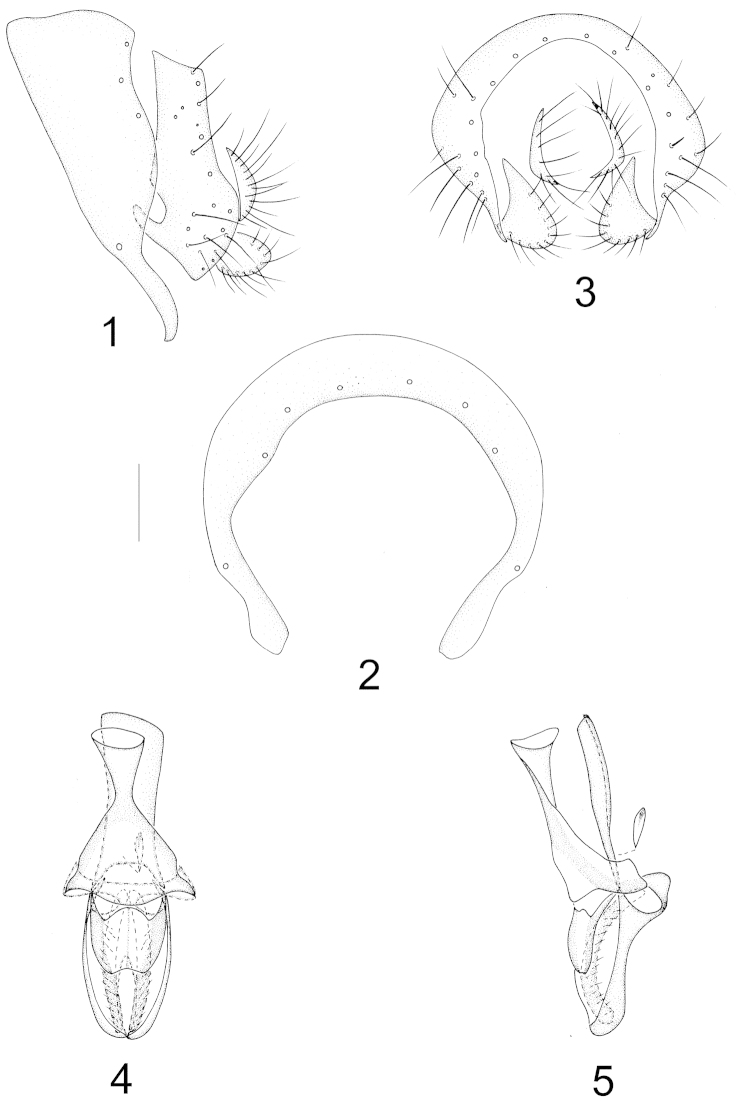
Minettia (Minettiella) atratula (Meijere, 1924). Male. **1** syntergosternite and epandrium, lateral view **2** syntergosternite, anterior view **3** epandrial complex, posterior view **4** aedeagal complex, ventral view **5** aedeagal complex, lateral view. Scale bar = 0.1 mm.

Abdomen with sparse brownish gray pruinosity. Male genitalia (Figs 1–5): syntergosternite semicircular with three pairs of dorsal setulae, epandrium narrow basally and broad apically, with a deep concave on anterior ventral margin and a digitiform anterior process, triangular apically in lateral view; surstylus separated from epandrium, elliptical in lateral view but crescent-shaped in posterior view; hypandrium nearly Y-shaped; postgonite forming a complete sclerite, with a median ridge; aedeagus consisting of a pair of clavate inner sclerites with short setulae and a pair of dorsolateral concaves, rounded apically; height of aedeagal apodeme nearly as long as aedeagus and broad in ventral view. Female terminalia (Figs 31–32): sternite 8 with a brown U-shaped spot, spermathecae 2+1, elliptical.

##### Distribution.

China (Hainan, Taiwan), Indonesia (Sumatra), Vietnam.

#### Minettia (Minettiella) bawanglingensis sp. n.

Taxon classificationAnimaliaDipteraLauxaniidae

http://zoobank.org/2742238E-F11B-4A8C-BDEF-85B255376811

Figs 6 –10 , 33 –34

##### Type material.

Holotype: ♂ (CAUC), CHINA, Hainan: Changjiang, Bawangling National Natural Reserve, Dong’er station, 1000 m, 24. v. 2007, Kuiyan Zhang. Paratype: 1♀ (CAUC), CHINA, Hainan: as holotype.

##### Etymology.

The new species is named after the type locality Bawangling National Natural Reserve.

##### Diagnosis.

Arista pubescent, with longest rays about 1/4 height of 1st flagellomere. Mesonotum with 0+2 *dc*, *acr* in 6 rows, two pairs of long *acr*. Mid femur with 3 *a*.

##### Description.

MALE. Body length 4.4 mm, wing length 4.2 mm. FEMALE. Body length 4.0 mm, wing length 3.9 mm.

Head. Frons slightly concaved with yellow anterior margin. Gena about 1/6 eye height. Antenna blackish brown, 1st flagellomere yellow at base, and 1st flagellomere nearly 1.6 times longer than high; arista pubescent, with longest rays about 1/4 height of 1st flagellomere. Proboscis black with dark yellow at apex and palpus black.

Thorax with brownish gray pruinosity. Mesonotum 0+2 *dc* (anterior *dc* far behind transverse scutal suture), *acr* in 6 rows; two pairs of long *acr* present in front of *prsc*, *prsc* slightly shorter than 1st post-sutural *dc*. Legs: tibia black with yellow at base, mid and hind tarsi yellow. Fore femur with 4 *pv* and 8 *pd*, fore tibia with 1 short preapical *ad* and 1 short *apv*. Mid femur with 3 *a* and 1 *app*; mid tibia with 1 strong preapical *ad* and 1 strong *apv*. Hind femura with 1 weak preapical *ad*, hind tibia with 1 weak preapical *ad* and 1 short *apv*. Wing with costa with 2nd (between R_1_ and R_2+3_), 3rd (between R_2+3_ and R_4+5_) and 4th (between R_4+5_ and M_1_) sections in proportion of 7:1.3:1; *r-m* beyond middle of discal cell; ultimate and penultimate sections of M_1_ in proportion of 1:1; ultimate section of CuA_1_ about 1/7 of penultimate section.

**Figures 6–10.**
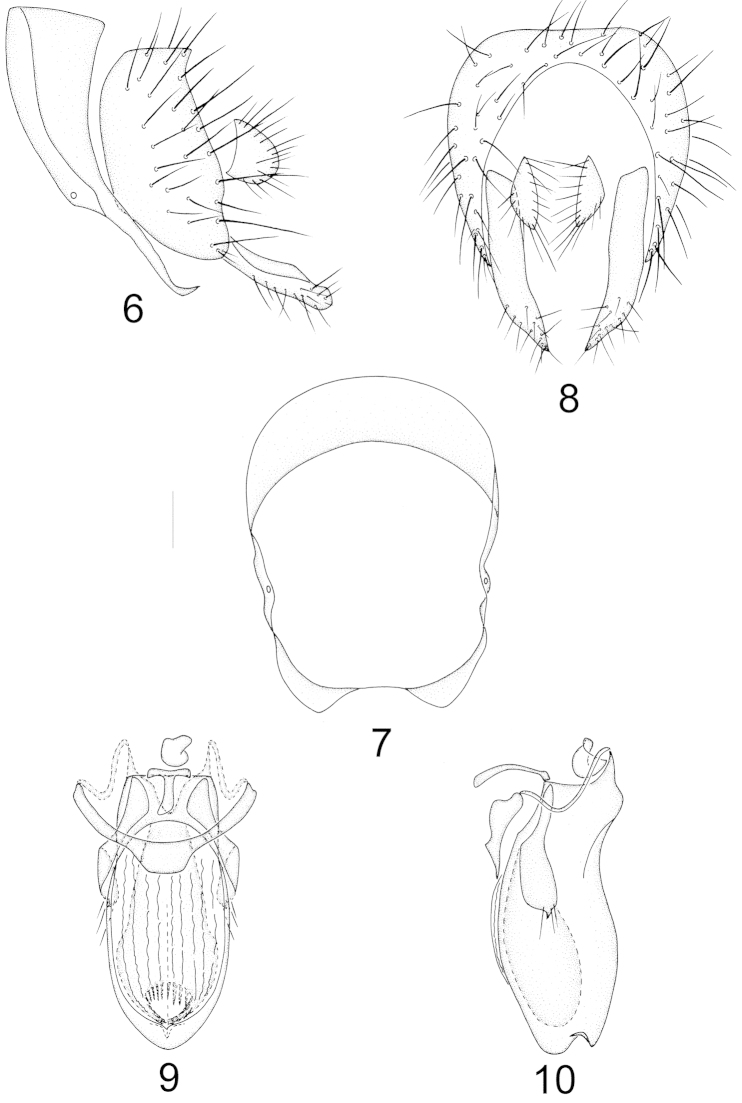
Minettia (Minettiella) bawanglingensis sp. n. Male. **6** syntergosternite and epandrium, lateral view **7** syntergosternite, anterior view **8** epandrial complex, posterior view **9** aedeagal complex, ventral view **10** aedeagal complex, lateral view. Scale bar = 0.1 mm.

Abdomen with sparse brownish gray pruinosity. Male genitalia (Figs 6–10): syntergosternite circular with a weak ventral bridge, epandrium with a tiny subapical concave, surstylus separated from epandrium, consisting of a long claviform process with setulae in lateral view and with a long triangular process in posterior view; hypandrium slender, projecting medially and nearly V-shaped; postgonite contorting, consisting of an acute triangular apical process and three apical setae; aedeagus columnar, truncate basally and blunt rounded apically, consisting of a triangular dorsal process subapically in lateral view and a black bottle-shaped inner process with an elliptical incision and many spiculate processes in ventral view; aedeagal apodeme narrow and short, projecting forwards, nearly right angle between hypandrium and aedeagus. Female terminalia (Figs 33–34): sternite 7 trapeziform with long setae on posterior margin, sternite 8 semicircular with three pairs of long setae on posterior margin and sternite 9 with a groove; spermathecae 2+1, round and all stems leading to the three spermathecae narrow apically.

##### Remarks.

The new species is very similar to Minettia (Minettiella) dolabriforma from the Palaearctic region in the following characteristics: mesonotum 0+2 *dc*, legs black with base of tibia yellow and mid and hind tarsi yellow, but it can be separated from the latter in the following characteristics: surstylus consisting of a long claviform process with setulae in lateral view and with a long triangular process in posterior view; hypandrium projecting medially and nearly V-shaped; aedeagus consisting of a triangular dorsal process subapically in lateral view and a black, bottle-shaped inner process with an elliptical incision and many speculate processes in ventral view. In Minettia (Minettiella) dolabriforma, the surstylus has a spatulate process, narrow ventrally and pointed at apex in lateral view; the hypandrium is U-shaped; the aedeagus is pointed dorsoapically with a lobate dorsal process in lateral view, and is spinulose and brownish-striated on the median ventral membrane in ventral view (Sasakawa 1995). The new species is also similar to Minettia (Minettiella) atrata from Indonesia (Java) in mesonotum with 0+2 *dc* and a pair of long *acr* present in front of *prsc*, but it can be separated from the latter in the arista having microscopic setulae, and the mid and hind tarsi being yellow. In Minettia (Minettiella) atrata, the arista is short plumose and only the hind tarsi are yellow (Meijere 1910).

##### Distribution.

China (Hainan).

#### Minettia (Minettiella) clavata sp. n.

Taxon classificationAnimaliaDipteraLauxaniidae

http://zoobank.org/A727B963-F589-4B19-910C-FC9F3D0BE476

**Figures 11–15.**
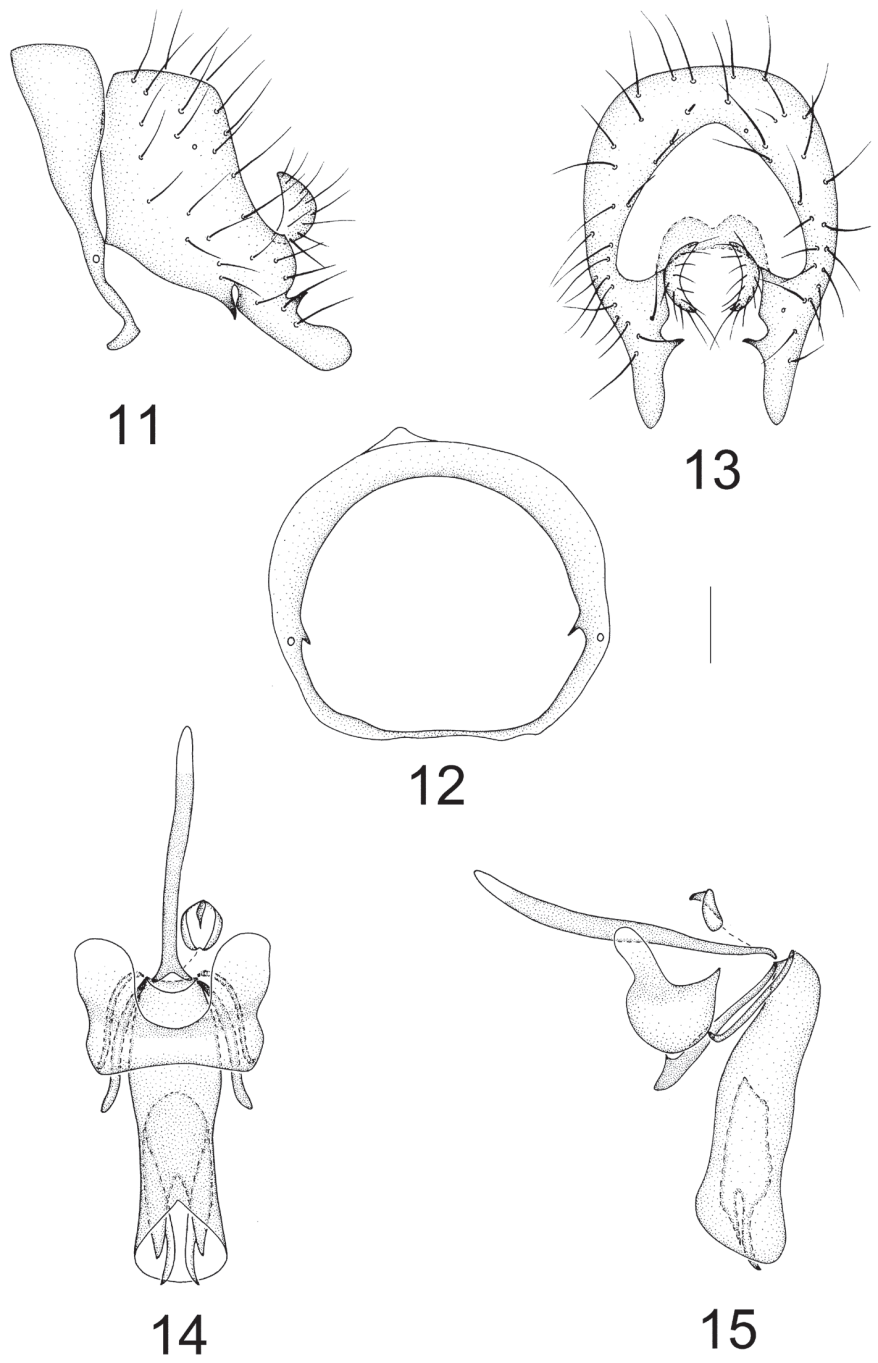
Minettia (Minettiella) clavata sp. n. Male. **11** syntergosternite and epandrium, lateral view **12** syntergosternite, anterior view **13** epandrial complex, posterior view **14** aedeagal complex, ventral view **15** aedeagal complex, lateral view. Scale bar = 0.1 mm.

Figs 11 –15 , 35 –36

##### Type material.

Holotype: ♂ (CAUC), CHINA, Hubei: Shennongjia National Natural Reserve, Pingqian, 1650 m, 26. VII. 2007, Qifei Liu. Paratypes: 4 ♂♂, 1♀ (CAUC), CHINA, Hubei: Shennongjia National Natural Reserve, Pingqian, 1650 m, 27. vii. 2007, Qifei Liu; 4♀ (CAUC), CHINA, Hubei: Shennongjia National Natural Reserve, Pingqian, 1650 m, 25. vii. 2007, Qifei Liu.

##### Etymology.

Latin, *clavata*, meaning clavate; referring to the club-like surstylus; a feminine adjective.

##### Diagnosis.

Antenna yellow with 1st flagellomere brown on apical 2/3; arista plumose, with longest rays slightly shorter than height of 1st flagellomere. Mesonotum 0+3 *dc*, anterior *dc* weak, hair-like; *acr* in 6 rows. All tibiae black with pale yellow at base; fore tarsi black, mid and hind tarsi dark yellow. Mid femur with 5 *a*.

##### Description.

MALE. Body length 2.9–3.7 mm, wing length 3.2–3.8 mm. FEMALE. Body length 3.0–3.3 mm, wing length 3.3–3.7 mm.

Head. Face slightly shining. Frons with yellow anterior margin. Gena about 1/6 eye height. Antenna yellow with brown on apical 2/3 of 1st flagellomere, 1st flagellomere nearly 1.7 times longer than high; arista plumose, with longest rays slightly shorter than height of 1st flagellomere. A blackish brown rectangular spot present between eye and base of antenna. Proboscis and palpus black.

Thorax with brownish gray pruinosity. Mesonotum 0+3 *dc* (anterior *dc* weak, hair-like, far behind transverse scutal suture), *acr* in 6 rows; *prsc* longer than 1st post-sutural *dc*; anepisternum with setulae on lower margin. Legs: all tibiae black with pale yellow at base; fore tarsi black, mid and hind tarsi dark yellow. Fore femur with 4 *pv* and 6 *pd*, fore tibia with 1 short preapical *ad* and 1 short *apv*. Mid femur with 5 *a* and 1 *app*; mid tibia with 1 strong preapical *ad* and 1 strong *apv*. Hind tibia with 1 weak preapical *ad* and 1 short *apv*. Wing with costa with 2nd (between R_1_ and R_2+3_), 3rd (between R_2+3_ and R_4+5_) and 4th (between R_4+5_ and M_1_) sections in proportion of 4:1.7:1; *r-m* at middle of discal cell; ultimate and penultimate sections of M_1_ in proportion of 1:1.1; ultimate section of CuA_1_ about 1/5 of penultimate section.

Abdomen shining. Male genitalia (Figs 11–15): syntergosternite circular with an inner tooth near spiracle, epandrium nearly rectangular; surstylus fused with epandrium, clavate with a triangular basal process, a projecting apical process, a small acute ventroapical process and a tiny incision in lateral view; hypandrium nearly U-shaped, hypandrial apodeme distinct; postgonite slender, broad apically in lateral view; aedeagus columnar and rounded apically, with a slight incision; aedeagal apodeme long, slightly shorter than aedeagus. Female terminalia (Figs 35–36): sternite 7 trapeziform, furcating apically with long setae; sternite 8 pale yellow, nearly columnar with a small apical incision; spermathecae 2+1, round.

##### Remarks.

The new species is very similar to Minettia (Minettiella) plurifurcata sp. n. (see discussion under Minettia (Minettiella) plurifurcata sp. n.) from China (Hubei). This new species is also similar to Minettia (Minettiella) atrata from Indonesia (Java) in having the arista short plumose and the mesonotum with *acr* 6 rows, and without other strong *acr* in front of strong *prsc*, but it can be separated from the latter by the mid and hind tarsi being yellow. In Minettia (Minettiella) atrata, only the hind tarsi are yellow (Meijere 1910; Malloch 1929).

##### Distribution.

China (Hubei).

#### Minettia (Minettiella) plurifurcata sp. n.

Taxon classificationAnimaliaDipteraLauxaniidae

http://zoobank.org/CAF5D321-6C62-41A3-A45F-B3F1FE218BAE

Figs 16 –20

##### Type material.

Holotype: ♂ (CAUC), CHINA, Hubei: Shennongjia National Natural Reserve, Pingqian, 1650 m, 26. vii. 2007, Qifei Liu.

##### Etymology.

Latin, *pluri*-, meaning many, and *furcata*, meaning forked; referring to the aedeagus with forked and acute processes in different lengths; a feminine adjective.

##### Diagnosis.

Arista plumose, with longest rays slightly shorter than height of 1st flagellomere. Thorax with whitish gray pruinosity, sparse on anterior 1/2 and dense on posterior 1/2. Mesonotum 0+3 *dc* (anterior *dc* far behind transverse scutal suture), *acr* in 6 rows. All tibiae pale yellow at base; fore tarsi black, mid and hind tarsi dark yellow. Mid femur with 4 *a*.

##### Description.

MALE. Body length 3.7 mm, wing legth 4.2 mm.

Head. Frons with yellow anterior margin. Gena about 1/7 eye height. Antenna yellow with brown on apical 2/3 of 1st flagellomere, 1st flagellomere nearly 2.0 times longer than high; arista plumose, with longest rays slightly shorter than height of 1st flagellomere. A black round spot present between eye and base of antenna. Proboscis and palpus black.

Thorax with whitish gray pruinosity, sparse on anterior 1/2 and dense on posterior 1/2. Mesonotum 0+3 *dc* (anterior *dc* far behind transverse scutal suture), *acr* in 6 rows; *prsc* as long as 1st postsutural *dc*; anepisternum with setulae on lower margin. Legs: tibia black with pale yellow at base; fore tarsi black, mid and hind tarsi dark yellow. Fore femur with 5 *pv* and 8 *pd*, fore tibia with 1 short preapical *ad* and 1 short *apv*. Mid femur with 4 *a* and 1 *app*; mid tibia with 1 strong preapical *ad* and 1 strong *apv*. Hind tibia with 1 weak preapical *ad* and 1 short *apv*. Wing with costa with 2nd (between R_1_ and R_2+3_), 3rd (between R_2+3_ and R_4+5_) and 4th (between R_4+5_ and M_1_) sections in proportion of 5.2:4:1; *r-m* at middle of discal cell; ultimate and penultimate sections of M_1_ in proportion of 1:1.2; ultimate section of CuA_1_ about 1/5 of penultimate section.

**Figures 16–20.**
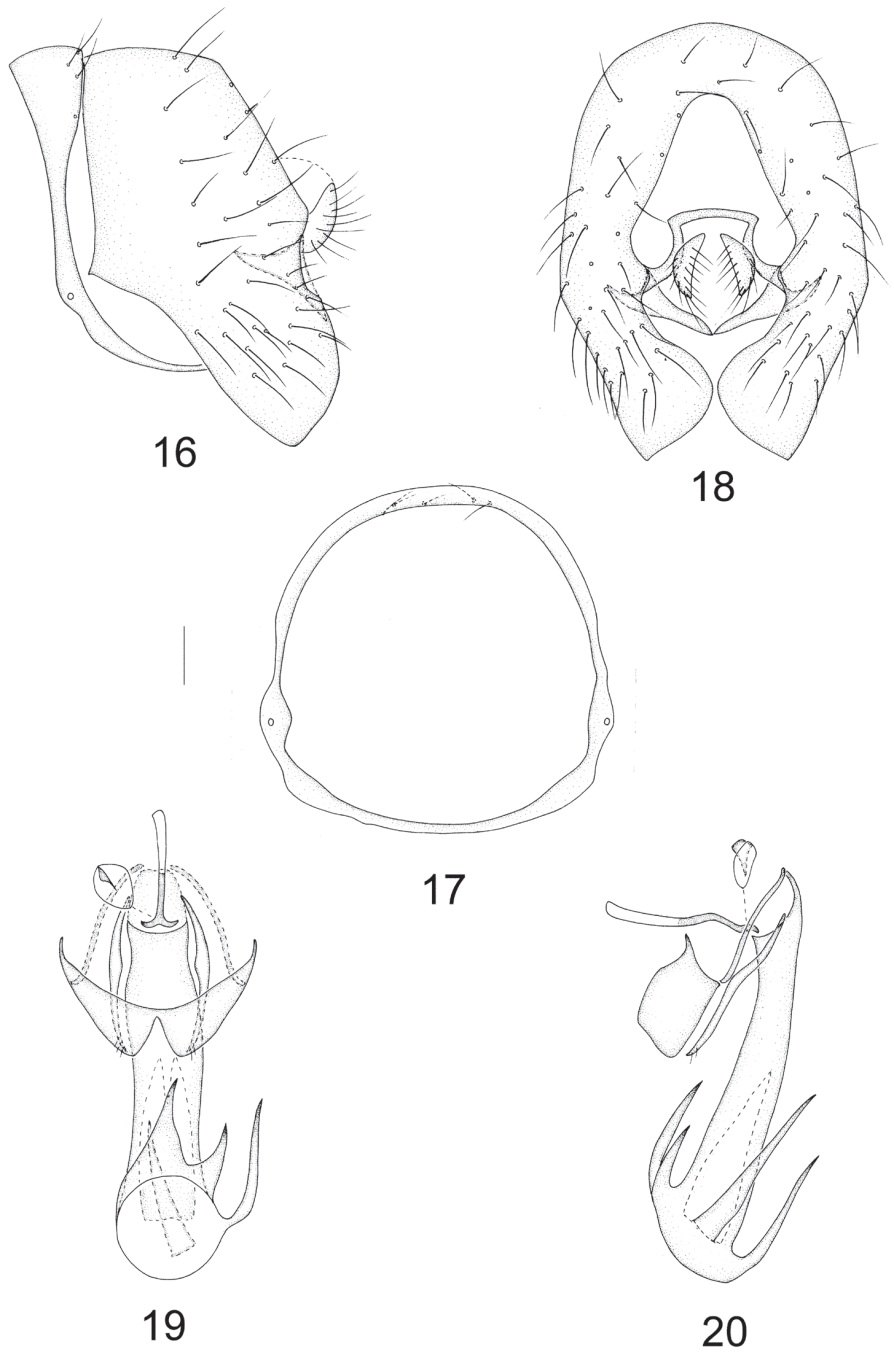
Minettia (Minettiella) plurifurcata sp. n. Male. **16** syntergosternite and epandrium, lateral view **17** syntergosternite, anterior view **18** epandrial complex, posterior view **19** aedeagal complex, ventral view **20** aedeagal complex, lateral view. Scale bar = 0.1 mm.

Abdomen with sparse whitish gray pruinosity. Male genitalia (Figs 16–20): syntergosternite circular with dorsal setulae, epandrium with a wide median incision on dorsal margin in lateral view; surstylus short subuliform and originating from inner side of epandrium in lateral view and convergent apically in posterior view; hypandrium nearly W-shaped; postgonite slender, slightly curved with 2–3 short apical setulae; aedeagus slender, columnar and rounded apically, with furcated acute processes in different length; aedeagal apodeme short, as long as 1/3 length of aedeagus.

FEMALE. Unknown.

##### Remarks.

The new species is very similar to Minettia (Minettiella) clavata sp. n. from China (Hubei) in the following characteristics: *acr* in 6 rows; all femora black and tibiae black with pale yellow at base; fore tarsi black, mid and hind tarsi dark yellow; wing yellow at base, but it can be separated from the latter in the following characteristics: the mesonotum having whitish gray pruinosity, sparse on anterior half and dense on posterior half, and 0+3 *dc*, anterior *dc* strong; the surstylus being long triangular and originating from the inner side of the epandrium. In Minettia (Minettiella) clavata, the mesonotum has 0+3 *dc* with anterior *dc* weak, hair-like; the epandrium is nearly rectangular; the surstylus is fused with the epandrium, and claviform with a triangular basal process a projecting apical process, a small acute ventroapical process and a tiny incision in lateral view.

##### Distribution.

China (Hubei).

#### Minettia (Minettiella) sasakawai

Taxon classificationAnimaliaDipteraLauxaniidae

Shi, Wang & Yang, 2011

Sapromyza (Sapromyza) acrostichalis Sasakawa, 2001: 50. Type locality: Vietnam.
Minettia (Minettiella) sasakawai nom. n. (comb. n. preoccupied by *acrostichalis* (Sasakawa & Kozánek, 1995), a new name as a replacement of *acrostichalis* Sasakawa, 2001)
Minettia (Minettiella) sasakawai Shi, Wang & Yang, 2011: 80 (with figures).

##### Material examined.

CHINA, Hainan (CAUC): 5 ♂♂, Ledong, Jianfengling National Natural Reserve, Plant garden, 800 m, 8. v. 2008, Qifei Liu; 1 ♂, Ledong, Jianfengling National Natural Reserve, Sanfenqu, 800 m, 8. v. 2008, Qifei Liu; 2 ♂♂, Ledong, Jianfengling National Natural Reserve, Plant garden, 800 m, 18. v. 2006, Gang Yao; 3 ♂♂, 2 ♀♀, Changjiang, Bawangling National Natural Reserve, Donger station, 1000 m, 24–25. v. 2007, Junhua Zhang; 1 ♂, Baisha, Yinggeling National Natural Reserve, 2. iv. 2006, Hui Dong; 1 ♂, 1 ♀, Baisha, Yinggeling National Natural Reserve, Hongmao village, 430 m, 21–22. v. 2007, Kuiyan Zhang; 1 ♂, Baisha, Yinggeling National Natural Reserve, Hongmao village, 430 m, 21–22. v. 2007, Junhua Zhang; 1 ♀, Baisha, Yinggeling National Natural Reserve, Hongmao village, 430 m, 21. v. 2007, Yongjie Wang.

##### Diagnosis.

Body black with brownish gray. Face and parafacial black flat with dense whitish gray pruinosity. Antennal 1st flagellomere blackish brown except yellow at base. Mesonotum 0+2 *dc* (anterior *dc* far behind transverse scutal suture), *acr* in 4 rows; a pair of long *acr* present in front of *prsc*. Legs mostly black except fore basal tarsus dark yellow on basal 3/4 and mid and hind tarsi dark yellow. Basal part of wing yellow. Abdomen shining black with sparse brownish gray pruinosity.

##### Redescription.

MALE. Body length 3.2–4.1 mm, wing length 3.4–4.4 mm. FEMALE. Body length 3.4–3.7 mm, wing length 3.6–3.9 mm.

Head black. Frons shining black except yellow, slightly concaved anterior margin; *oc* slightly longer than anterior *or*. Gena about 1/6 height of eye. Antenna scape and pedicel yellowish brown, 1st flagellomere blackish brown except yellow on basal part, 1st flagellomere nearly 1.8 times longer than high; arista with microscopic setulae. Proboscis black except yellow at apex; palpus black.

Thorax black with brownish gray pruinosity. Mesonotum with 0+2 *dc* (anterior *dc* far behind transverse scutal suture), *acr* in 4 rows; a pair of long *acr* present in front of *prsc*, *prsc* nearly as long as 1st postsutural *dc*; 1 *ia*, 1 *kepst*. Legs with fore basal tarsus dark yellow on basal 3/4 and mid and hind tarsi dark yellow. Fore femur with 5 *pv*, 8 *pd*, fore tibia with 1 long preapical *ad* and 1 short *apv*. Mid femur with 4 *a* and 1 *app*; mid tibia with 1 strong preapical *ad*, 1 strong *apv*. Hind femura with 1 weak preapical *ad*, hind tibia with 1 weak preapical *ad* and 1 short *apv*. Wing: costa with 2nd (between R_1_ and R_2+3_), 3rd (between R_2+3_ and R_4+5_) and 4th (between R_4+5_ and M_1_) sections in proportion of 6:1.7:1; *r-m* at middle of discal cell; ultimate and penultimate sections of M_1_ in proportion of 1:1.1; ultimate section of CuA_1_ about 1/6 of penultimate section.

Abdomen black with sparse brownish gray pruinosity. Male genitalia: protandrium semicircular (with a weak ventral bridge in a few specimens), narrow under spiracle; epandrium slender, with dorsal setae in lateral view; surstylus separated from epandrium, slender, narrow apically with a falcate apical process in lateral view; hypandrium very narrow at middle and broad on two lateral sides; postgonite columnar with two long setae; aedeagus acute apically with a tiny incision in ventral view but oblique truncate apically in lateral view; aedeagal apodeme nearly as long as aedeagus. Female terminalia: tergite 6 with a pair of long dorsal setae on posterior margin; spermathecae 2+1, round.

##### Distribution.

China (Hainan), Vietnam.

##### Remarks.

The species is very similar to Minettia (Minettiella) tianmushanensis sp. n. from Zhejiang (see Minettia (Minettiella) tianmushanensis sp. n.). The new species is also similar to Minettia (Minettiella) atrata from Indonesia (Java) in the following characters: mesonotum with 0+2 *dc* and a pair of long *acr* present in front of *prsc*, but it can be separated from the latter by the arista having microscopic setulae, the mesonotum having the *acr* in 4 rows, and the legs mostly black except the fore basal tarsus dark yellow on basal 3/4 and the mid and hind tarsi dark yellow. In Minettia (Minettiella) atrata, the arista is short plumose, the mesonotum has *acr* in 6 rows and the hind tarsi is yellow (Meijere 1910).

#### Minettia (Minettiella) spinosa sp. n.

Taxon classificationAnimaliaDipteraLauxaniidae

http://zoobank.org/0AB04092-C12C-4688-9F84-A5B564C66D9C

**Figures 21–25.**
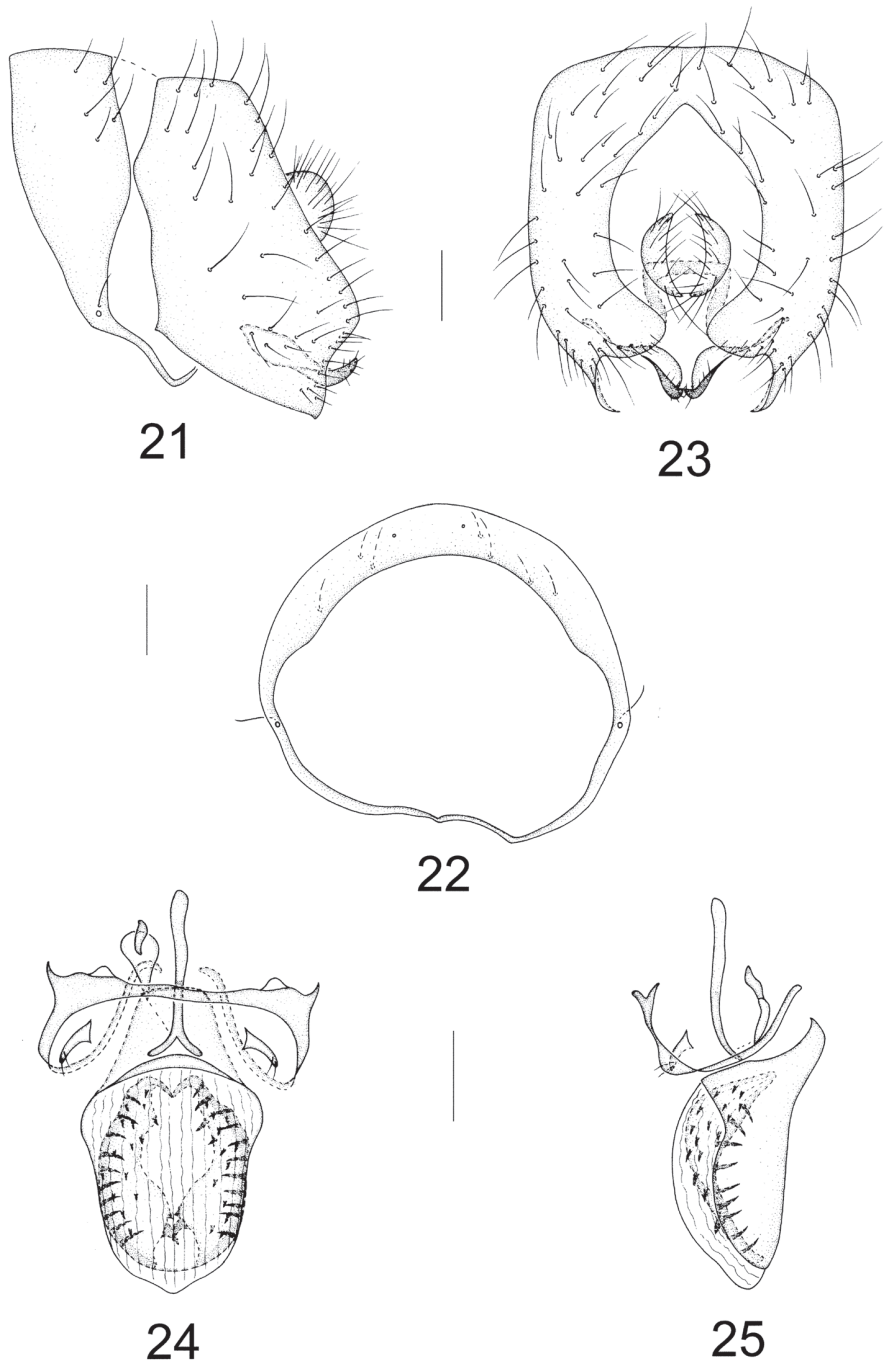
Minettia (Minettiella) spinosa sp. n. Male. **21** syntergosternite and epandrium, lateral view **22** syntergosternite, anterior view **23** epandrial complex, posterior view **24** aedeagal complex, ventral view **25** aedeagal complex, lateral view. Scale bar = 0.1 mm.

Figs 21 –25 , 39 –40

##### Type material.

Holotype ♂ (CAUC), CHINA, Hubei: Shennongjia National Natural Reserve, Pingqian, 1650 m, 25. vii. 2007, Qifei Liu. Paratypes: 4 ♂♂, 6 ♀♀ (CAUC), CHINA, Hubei: as holotype; 6 ♂♂, 5 ♀♀ (CAUC), CHINA, Hubei: Shennongjia National Natural Reserve, Pingqian, 1650 m, 27. vii. 2007, Qifei Liu.

##### Etymology.

Latin, *spinosa*, meaning spinose or with spinules; referring to the aedeagus with many short ventral spinules; a feminine adjective.

##### Diagnosis.

Face with a yellow triangular median spot or only slightly yellow in center of face. Arista with microscopic setulae, longest rays shorter than 1/3 height of 1st flagellomere. All tibiae dark yellow and tarsi dark yellow with tarsomeres 3–5 pale brown (apex of fore tibia pale brown in a few specimens); mid femur with 3 *a*. Wing black at base.

##### Description.

MALE. Body length 3.4–4.0 mm, wing length 3.9–4.4 mm. FEMALE. Body length 3.5–4.1 mm, wing length 4.3–4.6 mm.

Head. Face with a yellow triangular median spot or only slightly yellow in center of face, and parafacial brown with dense whitish gray pruinosity. Frons with yellow anterior margin (sometimes with a wide yellow median stripe in a few of specimens). Gena about 1/6 eye height. Antenna yellow with pale brown on apical 2/3 of 1st flagellomere, 1st flagellomere nearly 1.7 times longer than high; arista with microscopic setulae, longest rays shorter than 1/3 height of 1st flagellomere. An indistinct brown spot present between eye and base of antenna. Proboscis black with dark yellow at apex and palpus black.

Thorax with sparse whitish gray pruinosity. Mesonotum 0+3 *dc* (anterior *dc* far behind transverse scutal suture), *acr* in 6 rows; *prsc* slightly longer than 1st post-sutural *dc*. Scutellum with dense yellowish brown pruinosity. Legs: all tibiae dark yellow and tarsi dark yellow with tarsomeres 3–5 pale brown (apex of fore tibia pale brown in a few specimens). Fore femur with 5 *pv* and 8 *pd*, fore tibia with 1 short preapical *ad* and 1 short *apv*. Mid femur with 3 *a* and 1 *app*; mid tibia with 1 strong preapical *ad* and 1 strong *apv*. Hind femura with 1 weak preapical *ad*, hind tibia with 1 weak preapical *ad* and 1 short *apv*. Wing slightly yellow with black at base; costa with 2nd (between R_1_ and R_2+3_), 3rd (between R_2+3_ and R_4+5_) and 4th (between R_4+5_ and M_1_) sections in proportion of 11:1.5:1; *r-m* at middle of discal cell; ultimate and penultimate sections of M_1_ in proportion of 1:1.2; ultimate section of CuA_1_ about 1/6 of penultimate section.

Abdomen with sparse whitish gray pruinosity. Male genitalia (Figs 21–25): syntergosternite circular with a setula near spiracle, epandrium with a small subapical incision in lateral view and acute apically in posterior view, and surstylus contorting with apical setulae and originating from inner side of epandrium in lateral view; hypandrium slender, nearly H-shaped and hypandrial apodeme indistinct; pregonite tiny, slightly curved with two setulae; aedeagus broad, with dorsal sclerite round apically, membranous parts beyond apex of dorsal sclerite with short spinules and a pair of long hook-like basal processes; aedeagal apodeme slender. Female terminalia (Figs 39–40): sternite 7 rectangular, slightly concave on prosterior margin, sternite 8 semicircular with a pair of processes on anterior margin and a wide groove between processes; spermathecae 2+1, round.

##### Remarks.

The new species is similar to Minettia (Minettiella) plurifurcata sp. n. from Hubei in the following characteristics: mesonotum 0+3 *dc*, *acr* in 6 rows; fore femur with 5 *pv* and 8 *pd*, but it can be separated from the latter in the epandrium having a small subapical incision in lateral view and acute apically in posterior view and the surstylus being contorting with apical setulae in lateral view. In Minettia (Minettiella) plurifurcata, the epandrium has a wide median incision on the dorsal margin in lateral view and the surstylus is short subuliform in lateral view and convergent apically in posterior view.

##### Distribution.

China (Hubei).

#### Minettia (Minettiella) tianmushanensis sp. n.

Taxon classificationAnimaliaDipteraLauxaniidae

http://zoobank.org/9FE6AC20-3358-4C00-BBEC-98F53FCF9517

Figs 26 –30 , 41 –43

##### Type materials.

Holotype ♂ (CAUC), CHINA, Zhejiang: Lin’an, Tianmushan National Natural Reserve, 19. vii. 2007, Yajun Zhu. Paratypes: 5♂♂, 3♀♀ (CAUC), Zhejiang: as holotype; 1♂, 1♀ (CAUC), CHINA, Zhejiang: Lin’an, Tianmushan National Natural Reserve, Dajinggu, 20. vii. 2007, Yajun Zhu.

##### Etymology.

The new species is named after the type locality Tianmushan National Nature Reserve, Zhejiang Province

##### Diagnosis.

Frons slightly upturned with yellow anterior margin and short brownish yellow median stripe. Antenna brown with 1st flagellomere yellow at base; arista pubescent, with longest rays about 1/3 height of 1st flagellomere. Mesonotum 0+2 *dc*, *acr* in irregular 4 rows. Mid tarsi dark yellow and basitarsus with 1 subbasal *pv*, and hind tarsi dark yellow.

##### Description.

MALE. Body length 3.4–3.7 mm, wing length 3.4–3.6 mm. FEMALE. Body length 3.2–3.5 mm, wing length 3.3–3.6 mm.

Head. Frons with yellow anterior margin, slightly upturned and short brownish yellow median stripe. Gena about 1/10 eye height. Antenna brown with 1st flagellomere yellow at base and 1st flagellomere nearly 1.8 times longer than high; arista pubescent, longest rays about 1/3 height of 1st flagellomere. Proboscis and palpus blackish brown.

Thorax with brownish gray pruinosity. Mesonotum with 0+2 *dc*, *acr* in irregular 4 rows, a pair of long *acr* in front of *prsc*, *prsc* longer than 1st postsutural *dc*. Legs: tibia black, fore tarsi black, mid and hind tarsi dark yellow. Fore femur with 4–5 *pv* and 8 *pd*, fore tibia with 1 long preapical *ad* and 1 short *apv*. Mid femur with 3–4 *a* and 1 *app*; mid tibia with 1 strong preapical *ad* and 1 strong *apv*, mid basitarsus with 1 subbasal *pv*. Hind femur with 1 preapical *ad*, hind tibia with 1 weak preapical *ad* and 1 short *apv*. Wing with costa with 2nd (between R_1_ and R_2+3_), 3rd (between R_2+3_ and R_4+5_) and 4th (between R_4+5_ and M_1_) sections in proportion of 5:1:1; *r-m* beyond middle of discal cell; ultimate and penultimate sections of M_1_ in proportion of 1:1.1; ultimate section of CuA_1_ about 1/6 of penultimate section.

**Figures 26–30.**
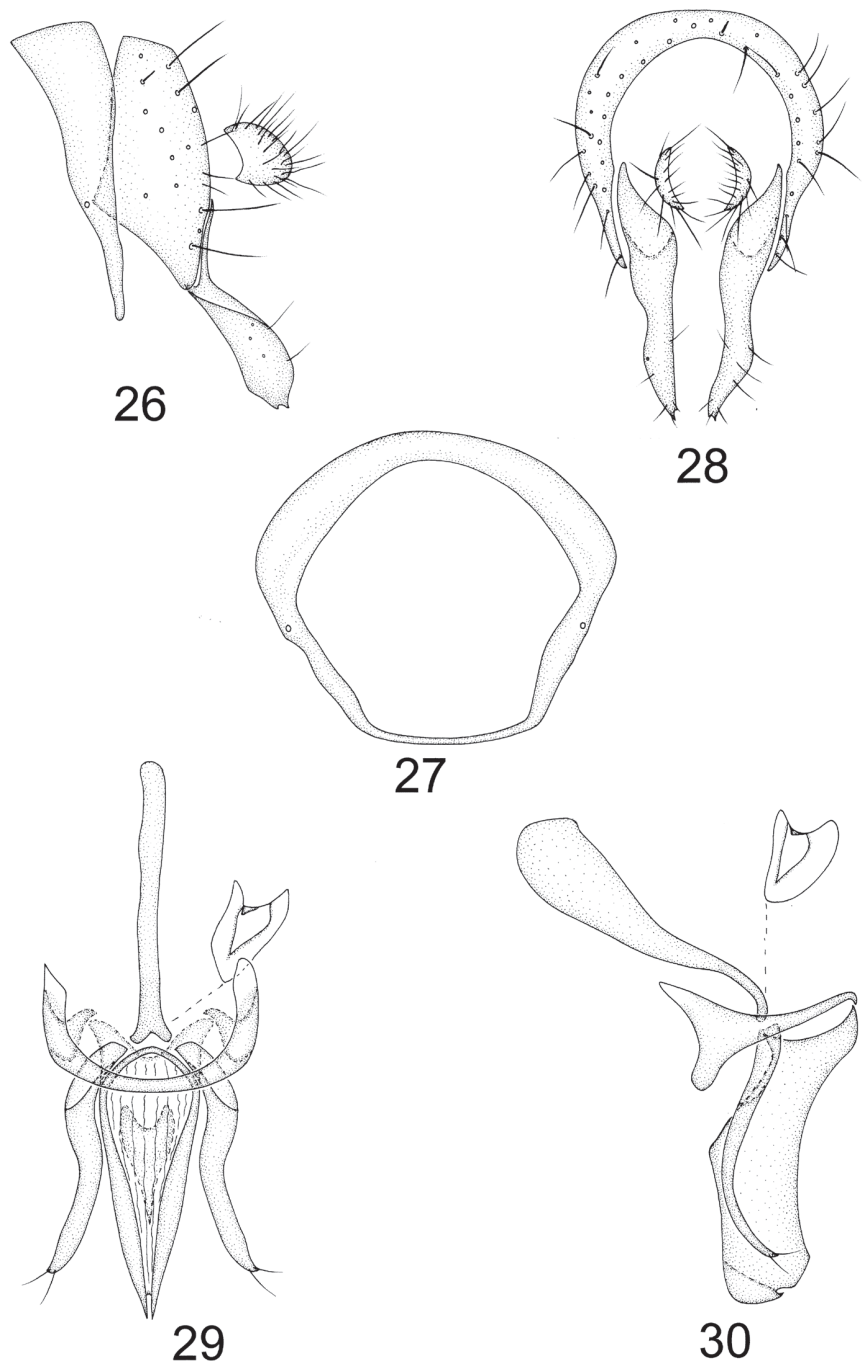
Minettia (Minettiella) tianmushanensis sp. n. Male. **26** syntergosternite and epandrium, lateral view **27** syntergosternite, anterior view **28** epandrial complex, posterior view **29** aedeagal complex, ventral view **30** aedeagal complex, lateral view. Scale bar = 0.1 mm.

Abdomen with sparse brownish gray pruinosity. Male genitalia (Figs 26–30): syntergosternite circular; epandrium slender, nearly rhombic and narrow apically; surstylus separated from epandrium, contorted claviform in lateral view and subuliform in posterior view with two acute apical teeth; hypandrium nearly U-shaped in ventral view, hypandrial apodeme distinct; postgonite S-shaped with two apical setulae in lateral view; aedeagus subuliform, narrow apically with a small incision in ventral view and wide rectangular, slightly projecting subapically with a small acute apical process in lateral view; aedeagal apodeme long, as long as height of aedeagus. Female terminalia (Figs 41–43): ternite 7 concave in lateral view, tergite 8 constricted medially; sternite 9 semicircular, sternite 8 slender, sternite 7 concave apically with a pair of triangular apical processes, curved outward; spermathecae 2+1, elliptical.

**Figures 31–43.**
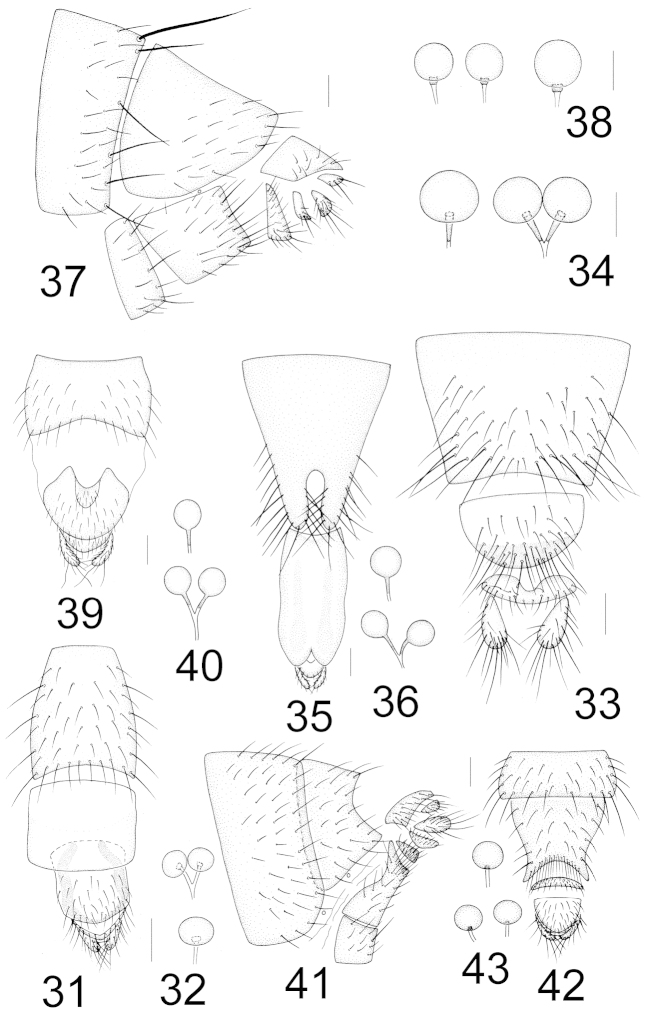
Female terminalia. Minettia (Minettiella) atratula (Meijere, 1924). **31** sternites 7–9, ventral view **32** spermathecae. Minettia (Minettiella) bawanglingensis sp. n. **33** sternites 7–9, ventral view **34** spermathecae. Minettia (Minettiella) clavata sp. n. **35** sternites 7–9, ventral view **36** spermathecae. Minettia (Minettiella) sasakawai Shi, Wang & Yang, 2011. **37** tergites and sternites 6–9, lateral view **38** spermathecae. Minettia (Minettiella) spinosa sp. n. **39** sternites 7–9, ventral view **40** spermathecae. Minettia (Minettiella) tianmushanensis sp. n. **41** tergites and sternites 6–9, lateral view **42** sternites 6–9, ventral view **43** spermathecae. Scale bar = 0.1 mm.

##### Remarks.

The new species is very similar to Minettia (Minettiella) sasakawai from China (Hainan) and Vietnam in the following characteristics: mesonotum with 0+2 *dc*, *acr* in 4 rows, and a pair of long *acr* present in front of *prsc*; wing pale yellow at base, but it can be separated from the latter in the syntergosternite being circular, the surstylus being contorting and claviform in lateral view and subuliform in posterior view with two acute apical teeth, and the female abdominal tergite 6 having no long dorsal setae on the posterior margin. In Minettia (Minettiella) sasakawai, the syntergosternite is semicircular and narrow under the spiracle; the surstylus is slender, narrow apically with a falcate apical process in lateral view and the female abdominal tergite 6 has a pair of long dorsal setae on the posterior margin.

##### Distribution.

China (Zhejiang).

### Key to the subgenera of *Minettia* and the species of the subgenus *Minettiella*

**Table:** 

1	Frons shining and face flat; arista pubescent; mesonotum with 0–1+2–3 *dc* and 0–1+2–4 long *acr*; katepisternum with 1 strong *kepst*; male genitalia: dorsal aedeagal sclerite present	subgenus ***Minettiella* Malloch ... 6**
–	Frons often dull and face slightly concave; arista pubescent or plumose; mesonotum with 0+3 *dc* and 0+2–3 long *acr*; katepisternum with 1 strong and 1 weak *kepst*; male genitalia: dorsal aedeagal sclerite absent (if dorsal aedeagal sclerite present, but no presutural *dc*)	**2**
2	Lower part of face with a distinct, slight and weakly round swelling on each side	**3**
–	Lower part of face without round swelling on each side	**4**
3	Basal part of wing black; arista long plumose, with longest rays longer than height of 1st flagellomere (rarely shorter than height of 1st flagellomere); male genitalia: ventral hypandrial appendages represented by two pairs of sclerites (including some Palaearctic and Oriental species)	subgenus ***Frendelia* Collin**
–	Basal part of wing yellow; arista short plumose, with longest rays as long as 1/2 height of 1st flagellomere; male genitalia: ventral hypandrial appendages represented by one pair of sclerites (such as *Minettia eoa* Shatalkin, 1992)	part of subgenus ***Scotominettia* Shatalkin**
4	Male genitalia: ventral hypandrial appendages represented by one pair of sclerites; arista short plumose, with longest rays as long as 1/2 height of 1st flagellomere (such as *Minettia austriaca* Hennig, 1951)	part of subgenus ***Scotominettia* Shatalkin**
–	Male genitalia: hypandrial appendages without representing by one pair of sclerites, often transverse bar-like, U-shaped or other shapes; arista plumose or pubescent	**5**
5	Arista with fine microscopic setulae, with longest rays as long as or shorter than 1/4 height of 1st flagellomere (rarely bare); wing yellow at base; male genitalia: aedeagal dorsal sclerite absent; postgonites fused dorsally forming a hood for aedeagus	subgenus ***Plesiominettia* Shatalkin**
–	Arista short to long plumose with longest rays longer than 1/3 height of 1st flagellomere; wing yellow or brown at base; male genitalia: aedeagal dorsal sclerite present (square, rectangular, triangular and trapeziform); postgonites separate	subgenus ***Minettia* Robineau-Desvoidy**
6	Mesonotum with 1+3 *dc*, *acr* in 2 rows, 1+3 long *acr*; male genitalia: epandrium narrow basally and broad apically, with a deep concavity on anterior ventral margin and a digitiform anterior process, triangular apically in lateral view; surstylus elliptical in lateral view (Figs 31–32)	**Minettia (Minettiella) atratula**
–	Mesonotum lacking a presutural *dc*, *acr* in 2–6 rows, without long *acr*; male genitalia: epandrium without deep concavity on anterior ventral margin and a digitiform anterior process in lateral view; surstylus not elliptical in lateral view	**7**
7	Mesonotum with 0+2 *dc*	**8**
–	Mesonotum with 0+3 *dc* (exceptionally Minettia (Minettiella) dolabriforma rarely with 0+3 *dc*, anteriormost *dc* is considerably smaller than usual, only half length of the second *dc*)	**12**
8	Mesonotum with *acr* in 6 rows (exceptionally Minettia (Minettiella) dolabriforma rarely with *acr* in 6 rows, a pair of *acr* long, just behind level of anterior *dc* and about two third length of *prsc*)	**9**
–	Mesonotum with *acr* in 4 rows	**10**
9	*acr* with two pairs of strong setae in front of one pair of *prsc* in dorsal view; arista pubescent, with longest rays about 1/4 height of 1st flagellomere; mid and hind tarsi yellow	**Minettia (Minettiella) bawanglingensis sp. n.**
–	*acr* with a pair of strong setae in front of one pair of *prsc* in dorsal view; arista plumose; only hind tarsi yellow	**Minettia (Minettiella) atrata**
10	Arista with microscopic setulae; male genitalia: surstylus narrow apically with a falcate apical process in lateral view	**Minettia (Minettiella) sasakawai**
–	Arista pubescent, with longest rays about 1/3 height of 1st flagellomere; male genitalia: surstylus wide apically with teeth or acute process in lateral view	**11**
11	Female sternite 9 rectangular, about three times as wide as long, and sternite 7 without triangular apical processes; male genitalia: surstylus with an acute process projecting forwards in lateral view	**Minettia (Minettiella) dolabriforma**
–	Female sternite 9 semicircular, sternite 7 with a pair of triangular apical processes (Figs 41, 42); male genitalia: surstylus contorted claviform in lateral view and subuliform in posterior view, with two acute apical teeth (Figs 26, 28)	**Minettia (Minettiella) tianmushanensis sp. n.**
12	Mesonotum with *acr* in 2 rows	**13**
–	Mesonotum with *acr* in 6 rows	**14**
13	Anepisternum with bluish grey pruinosity; mid and hind tibiae yellow	**Minettia (Minettiella) acrostichalis**
–	Anepisternum with whitish grey pruinosity; mid and hind tibiae yellow except blackish apical 1/4	**Minettia (Minettiella) coracina**
14	Mesonotum with brownish grey pruinosity, 1st postsutural *dc* weak, hair-like, *prsc* longer than 1st postsutural *dc*; male genitalia: surstylus fused with the epandrium, claviform with a triangular basal process, a projecting apical process, a small acute ventroapical process and a tiny incision in lateral view (Figs 11, 13)	**Minettia (Minettiella) clavata sp. n.**
–	Mesonotum with whitish grey pruinosity, 1st postsutural *dc* strong, *prsc* as long as or longer than 1st postsutural *dc*; male genitalia: surstylus articulated with epandrium, triangular or lobe-like with a single process	**15**
15	Face and parafacial with sparse whitish gray pruinosity; arista short plumose, longest rays slightly shorter than height of 1st flagellomere; mid femur with 4 *a*; male genitalia: epandrium with a wide median incision on dorsal margin in lateral view; surstylus short subuliform and originating from inner side of epandrium in lateral view and converging apically in posterior view (Figs 16, 18); female unknown	**Minettia (Minettiella) plurifurcata sp. n.**
–	Face with a yellow triangular median spot or only slightly yellow at middle of face, and parafacial with dense whitish gray pruinosity; arista with microscopic setulae, longest rays shorter than 1/3 height of 1st flagellomere; mid femur with 3 *a*; male genitalia: epandrium with a small subapical incision in lateral view and acute apically in posterior view, surstylus contorted with apical setulae and originating from inner side of epandrium in lateral view (Figs 21, 23); female sternite 8 semicircular with a pair of processes on anterior margin and a wide groove between processes (Fig. 39)	**Minettia (Minettiella) spinosa sp. n.**

## Supplementary Material

XML Treatment for Minettia (Minettiella) atratula

XML Treatment for Minettia (Minettiella) bawanglingensis

XML Treatment for Minettia (Minettiella) clavata

XML Treatment for Minettia (Minettiella) plurifurcata

XML Treatment for Minettia (Minettiella) sasakawai

XML Treatment for Minettia (Minettiella) spinosa

XML Treatment for Minettia (Minettiella) tianmushanensis

## Appendix

**Genus *Minettia* Robineau-Desvoidy, 1830**

**Subgenus *Minettiella* Malloch, 1929**

1. Minettia (Minettiella) acrostichalis (Sasakawa & Kozánek, 1995) (*Calliopum*). Oriental: North Korea.
2. Minettia (Minettiella) atrata (Meijere, 1910) (*Lauxania*). Oriental: Indonesia, Malaysia.
3. Minettia (Minettiella) atratula (Meijere, 1924) (*Lauxania*). Oriental: China (Taiwan, Hainan), Indonesia, Vietnam.
4. Minettia (Minettiella) bawanglingensis sp. n. Oriental: China (Hainan).
5. Minettia (Minettiella) clavata sp. n. Oriental: China (Hubei).
6. Minettia (Minettiella) coracina Shatalkin, 1993. Palaearctic: Russia.
7. Minettia (Minettiella) dolabriforma (Sasakawa & Kozánek, 1995) (*Calliopum*). Palaearctic: North Korea, Japan, Russia.
  *Minettia japonica* Sasakawa, in Sasakawa & Mitsui, 1995. Syn. n.
  *Minettiella elbergi* Shatalkin, 1996. Syn. n.
8. Minettia (Minettiella) plurifurcata sp. n. Oriental: China (Hubei).
9. Minettia (Minettiella) spinosa sp. n. Oriental: China (Hubei).
10. Minettia (Minettiella) sasakawai Shi, Wang & Yang, 2011. Oriental: China (Hainan), Vietnam.
  *Sapromyza acrostichalis* Sasakawa, 2001. [Homonym of *Calliopum acrostichalis* Sasakawa & Kozánek, 1995, when in *Minettia*]
11. Minettia (Minettiella) tianmushanensis sp. n. Oriental: China (Zhejiang).
